# Supplementary material for: CD36 and SR-B1 polymorphisms exhibit distinct association patterns in active and latent tuberculosis
Source: J Med Microbiol. 2025 Dec 18;74(12):002111. doi: 10.1099/jmm.0.002111 (PMC12713683; doi:10.1099/jmm.0.002111)
Supplement: Uncited Supplementary Material 1. [file jmm-74-02111-s001.pdf]

Supplementary Tables

Table S1. Inclusion and exclusion criteria applied while selecting study subjects.

Study Group	Inclusion criteria	Exclusion Criteria
**Active TB patients**	Newly diagnosed with TB	HIV infected*<br>Pregnant/breastfeeding women*
**TB Contacts**	Household contacts<br>Workplace contacts<br>Asymptomatic	Clinical suspicion of active TB<br>Blood relation with active TB patient
**Healthy Controls**	Doesn't show symptoms of TB	Diagnosed with TB<br>Have been in contact with TB patient/s

*These criteria were applied to exclude participants from all study groups. The age limit for all participants was ≥16 years.

Table S2. Sequence and properties of primers.

Gene	Mutation	Primer Sequence	Length (bp)	Tm (°C)
SR-B1	rs5888	**Outer forward:** ACACCCAAGTTCAAATCTGGCGTTGC	26	66.0
		**Outer reverse**: ATGGAGTCGGGGTGAAGTAAGGAACT	26	66.6
		**Inner forward:** CTCTCCCATCCTCACTTCCTCAACTCC	27	68.8
		**Inner reverse:** CTTCTGCCAGAACCGGGGCA	20	69.1
	rs4238001	**Outer forward:** CTCCCGCCCCAAAACGGAA	19	67.7
		**Outer reverse**: CAGCAGCCCCTCCCGAAGC	19	70.6
		**Inner forward:** CCAGGCGCGCAGACAGGA	18	68.9
		**Inner reverse:** GCGCTTTGGCGGAGCATCC	19	67.0
CD36	rs1761667	**Outer forward:** AAGGTCTGGTATCCACCTGTTTTCCT	26	64.7
		**Outer reverse**: AAGAGTTTTCATGAAGCTTCCCGC	24	61.9
		**Inner forward:** TTTTATTCATCTTTGCATGCCATCG	25	57.3
		**Inner reverse:** TCATACTCCAGGCTTTGAGCATTGT	25	61.9
	rs3211938	**Outer forward:** GAATAGTTCATGCTTGGCTATTGAGTTT	28	58.2
		**Outer reverse**: CACCATTCTTTCTTCTGCCCTAATTACT	28	60.4
		**Inner forward:** AAAATTATCTCAAAAAATTGTACATCACAG	30	53.5
		**Inner reverse:** TGCATTTGCTGATGTCTAGCACATCA	26	61.4

Table S3. PCR conditions for all single nucleotide polymorphisms.

Mutation	PCR conditions				
	**Initial denaturation**	**Denaturation**	**Annealing (35 cycles)**	**Extension**	**Final extension**
**rs5888**	95 °C for 5 min	95 °C for 1 min	68 °C for 1 min	72 °C for 1 min	72 °C for 10 min
**rs4238001**	95 °C for 5 min (for both first 10 cycles & 25 cycles)	95 °C for 1 min (for both first 10 cycles & 25 cycles)	65 °C (-1°C for every first 10 cycles) & 55 °C (for 25 cycles) 1 min	72 °C for 1 min (for both first 10 cycles & 25 cycles)	72 °C for 10 min
**rs1761667**	95 °C for 5 min	95 °C for 1 min	61 °C for 1 min	72 °C for 1 min	72 °C for 10 min
**rs3211938**	95 °C for 5 min	95 °C for 1 min	51 °C for 1 min	72 °C for 1 min	72 °C for 10 min
